# Transactions of the Fourth Annual Meeting of the American Association of Obstetricians and Gynecologists

**Published:** 1892-02

**Authors:** 


### Society Proceedings.

[*From The American Journal of Obstetrics.*]

### TRANSACTIONS OF THE FOURTH ANNUAL MEETING OF THE AMERICAN ASSOCIATION OF OBSTETRICIANS AND GYNECOLOGISTS.

HELD IN NEW YORK CITY, SEPTEMBER 17, 18, AND 19, 1891, AT THE ACADEMY OF MEDICINE.

(*Abstract.*)

(Continued from page 348.)

### SECOND DAY—MORNING SESSION.

THE President, DR. A. H. WRIGHT, of Toronto, in the chair.

Dr. M. B. WARD, of Topeka, Kan., read a paper on

#### REMOVAL OF THE UTERINE APPENDAGES, WITH RESULTS.

He preceded the report of his cases by saying that operation for removal of uterine appendages is truly missionary work in Kansas. He performed the second operation of this character done in the State, February, 1890.

He then made a brief report of fifteen cases, in which he gave a history of the patient before the operation, the character of the operation, and the results at the time of writing the paper.

Three deaths were recorded, his first, third, and eleventh cases terminating fatally. Only one of the three was caused by the operation, and in that case the previous condition was deplorable. The reports of the cases were interesting and the results gratifying. Some of the conditions were extremely grave before the operation, and yet recovery in most of the cases was satisfactory.

#### DISCUSSION.

Dr. JOSEPH PRICE, of Philadelphia: We all know how satisfactory the results have been of late with this class of cases in dispensary and in public practice. We also know how distressing the results have been among the more wealthy, where harm follows from the delay due to treatment by rest and every other known method before resorting to radical measures.

Our mortality among the rich varies between six and ten per cent. higher than among the poor, and there is no excuse for such a difference. A pus tube, an abscess in the pelvis, a pus accumulation in the axilla, under the deep tissue anywhere, should be removed. The treatment is precisely the same here. Good surgeons throughout the world will trephine for an accumulation of pus not larger than a hazelnut. Surely, if it holds good in brain surgery, it should hold good in pelvic accumulations. Again, just this state of affairs is common in all branches of abdominal surgery. It is so with gall stones and other hepatic accumulations. It is so notwithstanding we have had many years of special pleading, many voices crying in the wilderness—such men as Bantock, Keith, Tait, Thornton, and others pleading specially for early ovariotomy. We find large numbers of neglected cystomata, and the mortality remains higher than it should in that class of cases. He said that gonorrhea is unquestionably responsible for a majority of these cases. Gonorrhea is no longer treated by intelligent physicians. Every drug store in the land is a clap shop, probably as well in Boston as Philadelphia. It is a neglected disease. The sequelæ, in the male as well as in the female, are very much more marked than they were several years ago when the disease was treated by the educated physician.

Dr. E. W. Cushing, of Boston, said he appreciated and believed every word that Dr. Price had uttered. The bad cases that he had seen were the ones that had been neglected ; early operations had done well. Allowance must be made for the position of a man with his elders against him. It is dangerous for one to get too far ahead of the sentiment of the community. The weight of the teaching and the intent of every operator, as far as he can carry his people along with him, should be to operate early.

Dr. Mordecai Price, of Philadelphia, thought many of the delays were attributable to the cowardice of the surgeon. We all should know that when we approach a question where a life is involved, we should boldly consider every side, even though, if we operate and the life is lost, the community says that it was the operation. If we refuse to operate and death comes, we feel and know that it was but a consequence of our own cowardice. It is our business to go to these cases, and when we discover that pus exists, to remove it, and the sooner the better.

Dr. C. A. L. Reed, of Cincinnati, extended his congratulations to Dr. Ward for his good paper, which is certainly to be recognized as pioneer work in the great State of which he is a citizen. He was impressed with the idea that gonorrhea in woman is a very serious affair. It progressively invades the mucous tract, climbing the vaginal wall, climbing the endometrium, invading the sanctum sanctorum of the female anatomy, and producing its most serious ravages upon the uterine appendages.

Dr. Ward, closing the discussion, thanked the Fellows for their kind compliments. In the West it is extremely difficult to get consent to perform these operations except as a last resort.

Dr. Joseph Price, of Philadelphia, read a paper upon

### A CONSIDERATION OF EMMET'S LAST OPERATION FOR SO-CALLED LACERATION OF THE PERINEUM, OR PROLAPSE OF THE POSTERIOR WALL OF THE VAGINA FROM LOSS OF FASCIAL SUPPORT, OR MUSCULAR RELAXATION, OR TEAR.

At the meeting of the American Gynecological Society in 1883, when Dr. T. A. Emmet presented his paper on the Etiology of Perineal Laceration, with a New Method of Operation for its Repair, it was evident, in the words of an eminent gynecologist, that the members of the Society were very much at a loss to comprehend the steps of the operation as described by its illustrious originator. It is still not understood generally in Philadelphia, where at the present time we rarely hear of any one's performing the true Emmet operation unless it has been improved by some supposed modification. These modifications are for the most part meretricious. The Emmet operation seems to fulfil every indication for the restoration of the damaged perineum, far better than any other operation before or since devised, not excepting the so-called flap-splitting or Tait's operation, over which so much furore has lately been raised. The value of the operation has not been recognized, because the published reports of it have been so meagre and so little descriptive, excepting some explanations and modifications, that the very idea of the operation has not been grasped, except by those who, like the writer, have been fortunate enough to have Emmet personally demonstrate the operation and its advantages. Unfortunately, his original pamphlet was rather confusing than instructive and explanatory to those not already comprehending the operation. A drawing was necessary for the exact understanding of the successive steps of the operation. In several cases in which Emmet's operation was performed, the writer had the assistance of Dr. J. Madison Taylor, who has sketched the appearance of the various stages. The theory of the mechanism of laceration is original with Emmet. He believes that the damage that leads to the severe consequences of loss of support to the pelvic diaphragm is principally in the deep layers of the pelvic fasciæ, a separation of the fibers of those fasciæ from the outlet of the vagina. The soft parts of the vagina are crowded up in advance as the fetal head passes. When the head of the child is forced down upon the so-called perineal body, the perineal tissues are stretched transversely before the head appears at all at the vaginal outlet. The so-called perineal body is attached principally at the two rami of the ischia through the strong and almost inelastic ischio-perineal fascia. When the head, following the sacral curves, crowds down the vaginal tissues before it, it meets its almost entire resistance from a lack of extensibility in these ligaments. If the force is severe enough they will separate, not by a tear from the fourchette downward, but by a separation of those fibers within the mucous membrane from their insertion without and below the vaginal orifice. It is the pelvic fascia that supports the vagina, this fascia often separated from its attachment to the vagina, or laterally from one or both rami at the passage of the head during parturition. The tear at the orifice of the vagina is of infinitely less importance than is the rupture of the ischio-perineal fascia. These fasciæ preserve the proper resilience of the floor of the pelvis. This removed, the anterior and posterior walls of the vagina have lost their support, and they must prolapse through the now weakened vaginal ring. It is well known that rectocele occurs constantly in patients in whom the fourchette has not been torn. A vagino-perineal laceration may pass around the sphincter ani. It is easily shown that the perineum is a thin diaphragm, not a body of firm fascia, muscle, or connective tissue. The rupture, which may or may not have been submucous, is not in the median line, but has been more severe on one side than on the other. In Emmet's expressive illustration, the bag has lost its puckering string. The prolapse is evidently due to a want of resisting power on the pelvic floor. Other operations have been previously devised to meet the same indications, such as those of Simon, Hegar, Hildebrandt, Freund Bantock, Baker, Brown and Emmet's trefoil operation. Some of these restored the perineum so that it was even longer than before, but failed in its real intention, since little by little the rectocele again prolapsed through the narrowed outlet to its original condition. A great advance was made when the denudation was extended so as to include a portion of the rectocele and so restore by cicatricial healing the perineal body. The results were frequently anything but happy. The old operations aimed to replace the perineal body by a new structure built from the adjacent parts ; but when a perineum is lacerated it is not destroyed. If we can bring together the ends of the ruptured ischio-perineal ligaments, we will absolutely restore the supporting power of the perineum. Emmet's last operation aims at removing the superfluous vaginal mucous membrane at the vulvo-vaginal orifice, continuing at the same time the denudation along the line of the original rupture. As this line extends usually along the two sulci, the denudation assumes rudely the form of a crescent, whose convexity is the boundary of skin and mucous membrane of the vulva, while the concavity is marked by the summit of the projecting rectocele, and the cusps extend up the lateral deep sulci and posterior wall of the vagina. Finally he introduces sutures entirely within the vagina, beginning at the apex of a cusps and closing by bringing the two denuded edges of the cusp or sulcus, together. After the insertion of the crown stitch, one or more superficial stitches are sometimes necessary, according as there has been greater or less laceration. The essential landmarks of the operation—to-wit, the crest of the rectocele or its most projecting part, and the lateral mucous membrane at the highest point to which it is intended to carry the denudation—are not to be lost sight of or changed as the operation proceeds. After the sulci have been closed, when the crown stitch is introduced, the inside stitches entirely disappear. The after-treatment is simple—rest, catheterization if necessary. The bowels are to be kept easily open. The writer has yet to see the slightest inflammation—cellulitis or phlebitis—from the operation. The stitches are removed at the end of six or ten days, except those in the sulci. These latter are removed in the second week. This is best done by partially everting the vagina by a finger introduced into the rectum.

This operation most nearly approximates the natural perineum. The great degree of success attending the operation is alone sufficient to recommend it primarily in all lesions of the perineum.

DISCUSSION.

Dr. L. S. McMurtry, of Louisville, thought the importance of the subject increased by the great number of cases there are of perineal rupture coming up daily in the practice of gynecologists, and the large amount of imperfect work that is done in this branch of surgery, and the incongruous advice that is given to patients upon this subject. The general surgeon does a good deal of this work, and it is very often the case that the skin only is united, and in all probability the skin is attached away up the vulvo-vaginal fissure, higher than it was in the natural condition; and in many instances the physician, finding that the fourchette is not torn, assures the woman that her symptoms are due to nervous exhaust ion and that there is no laceration of the perineum. It has been demonstrated this morning that you may sew together the skin without restoring the pelvic floor, and you may have the pelvic floor entirely destroyed without any rupture of the skin externally. Those are points which have been emphasized in the paper, which are not appreciated generally by the profession in regard to these injuries. The great difficulty in regard to the popularity of Emmet's improved operation for restoring the pelvic diaphragm is that it is difficult to clearly appreciate the operation without seeing it executed, and that it is very difficult to do the operation without giving it thorough consideration.

Dr. W. H. Wathen, of Louisville, thought Dr. Price had made an important subject unusually interesting, because of the practical manner in which he had presented it, and because of the vast experience he had had in doing successful work in this department of surgery, which makes all he says practical. In the main he fully agreed with him in his ideas and in his suggestions as to the proper means of operating. In operations where the tissues are torn down near the sphincter, with the anterior rectal wall presenting down in the vagina, he was practically in accord with the suggestions of Dr. Price, excepting that the procedure might be simplified by splitting the tissues between the rectum and the vagina latterly instead of denuding the mucous membrane. The splitting process can be accomplished by an experienced operator in less than half the time of the denuding process, and with not less than half the inconvenience from hemorrhage. The cardinal principle which should control us in this operation is to expose the ends of the torn muscles and the ends of the torn fascia, superficial, middle, and deep, and a failure to expose all these so that they may be brought together and held in position necessarily results in a corresponding failure in the result of the operation.

We ought to impress upon the general practitioner and the general surgeon, as well as the specialist, the importance of attending to these cases wherever they are met, but, above all, we ought to impress upon the profession the necessity of attending to these cases when they occur. There is no operation of any magnitude in the whole range of gynecic surgery that has the element of simplicity so perfect and the universal results so attractive as in the operation for complete or incomplete laceration of the perineum, if done immediately after the accident has occurred.

Dr. J. F. W. Ross, of Toronto, believed that the denudation of the tissues outside is superfluous, but is necessary in the vagina, and that we can combine the operation of denuding in the vagina with flap-splitting in the perineum, exactly as Dr. Wathen had said.

Dr. Mordecai Price, of Philadelphia, spoke strongly in favor of the Emmet operation. He believed with Dr. Wathen that we have no business to know anything about secondary perineal work. It ought to be the business of the physician to have his materials with him, even if the case is a simple one, because accidents will occur in the best regulated households in that particular; and it ought to be his business to at once apply his sutures, because union is almost invariably by first intention in these primary operations, and there is no better way than to use the silkworm-gut sutures, passing them as Dr. Emmet recommends. The tear in nine cases out of ten is in the right or left sulcus.

Dr. H. T. Hanks, of New York, said he had been associated more or less intimately with Dr. Emmet in the Woman's Hospital for the last thirteen years, and had watched the development of the operation. He had been pleased to have the subject brought up for that very reason, because he has not only done good work in teaching us how to operate on a lacerated cervix, but has also given us some points in the operation on the perineum that we have not considered before, and which, when carried out to the letter as he has taught us, and as those of you who have seen him operate have been taught, give entirely satisfactory results. He believed the operation to be a successful one when well done, and done as Dr. Emmet had taught.

Dr. Joseph Price, of Philadelphia, closing the discussion, said: Some of us do the operation perhaps a little more than Dr. Emmet does—just a little more—but it all belongs to him. He taught us how to denude and everything that is good in plastic surgery.

Some of us go a little further than Dr. Emmet. Dr. Hanks has made a perfect illustration of Dr. Emmet's operation. Many of us differ in this particular. Dr. Emmet does not go as high in the sulci as some of his pupils with the denuding. Dr. Emmet makes a triangle with a tenaculum; we make it with a denudation, and go into the apex of that sulcus. We give the woman as much pelvic floor as possible. There are very serious doubts as to whether a flap-splitting operation ever restores a sphincter. I have yet to see the first one, and know at least half a dozen women in Philadelphia who have had the flap-splitting operation. They have incontinence of gas and feces when they have a diarrhea. I have seen men do it and absolutely miss, in their denudation and everything else, the dimples of the retracted sphincter, or half-moon. There are very few men in this country that can map out the sphincter in a perineal tear through the sphincter. Dr. Emmet, if you will follow him, will place his fingers on the sphincter and make you trace a hard, iron-like sphincter all the way round. He again has taught us all we know about sphincter tears that is worth knowing. Emmet's inside perineal operation with the sulci sutures closed is an operation which completes the woman's comfort. He need not put in those inside sutures, as far as the perfection of the operation and the comfort of the patient are concerned. The operation is complete when he completes his sulci work.

The President then delivered his annual address on

THE GENERAL AND SPECIAL MEDICAL SOCIETIES OF AMERICA.[1]

Dr. W. W. Seymour, of Troy, read a paper on

A CASE OF CHOLECYSTOTOMY AND CHOLELITHOTRITY; DEATH FROM "LA GRIPPE" THE TWENTY-FIRST DAY.

The gall bladder was incised and fine stones removed from it, and one stone in the common duct crushed by forceps applied to the duct. The crushing was done because the wound was so deep that the difficulties in the way of an exact suture of the duct in case of excision of the stone were enormous. At the autopsy, when death resulted from an intercurrent attack of grippe, the abdominal wound, save at the drainage opening, was firmly united, the adhesion of the gall-bladder perfect, and there was not the slightest evidence of suppuration, inflammation, or ecchymoses in

1. Published in full in the November (1891) number, page 193.

or about any abdominal organ. The inferences drawn were: 1. That in large, fat, and flabby bellies crushing is safer than excision with its risk of imperfect suture. 2. That excision should only be a method of election where an exact suture is beyond question. 3. That if there is any suspicion of injury to the duct, drainage should be inserted to the suspected point, and, if needed, the abdominal cavity protected by gauze tampons. 4. That in case of stones projecting into or overlying the duodenum, the duodenum may be incised and the stone, if projecting, broken up, or the duodenal portion incised and the stone crushed or delivered intact, as Dr. Charles McBurney has recently successfully done with a stone as large as a pigeon's egg.

Dr. A. Vander Veer, of Albany, read a paper entitled

### REPORT OF CASES OF CHOLECYSTOTOMY, WITH SPECIAL REFERENCE TO THE TREATMENT OF CALCULUS LODGING IN THE COMMON DUCT.

He said: In presenting this paper as a contribution to the surgery of the gall ducts, I shall refer somewhat to the surgery of the gall-bladder. Yet my chief desire is to get from you a discussion and your views regarding a line of treatment in cases where we find a contracted and perhaps almost obliterated gall-bladder, or where the common, cystic, or hepatic ducts are entirely closed by lodgment of a calculus, or stenosis from other causes. He then presented a group of three cases illustrating these points, in which he had operated twice successfully, and exhibited specimens removed in each. From his experience in these and other cases he offered the following:

This operation is not very difficult, and the results favorable beyond a doubt; but does not this method deter us in some cases from pushing ahead and relieving cases where practically no gall-bladder is to be found? Are we not too conservative at times where we have stenosis, severe adhesions, and apparently no gall-bladder present?

The intent of this paper is to deal only with those cases where there is an obstruction of the ductus communis choledochus, the cystic or hepatic ducts, from any cause whatever. Cholecystotomy, with suture of bladder walls to parietal wound, when the viscus is fairly well developed, is not a serious operation. Primary and secondary opening of the gall-bladder I mention here to emphasize the fact that I believe the latter is always to be condemned for the following reasons :

1. It is always necessary, before the closure of the abdominal wound, that the gall-bladder be opened and emptied, as it is often impossible to recognize conditions until then which will require manipulations not only within the gall-bladder, but also within the abdomen.

2. Prolonged obstruction in the intestinal portion of the common duct may lead to its dilatation, together with that of the gall-bladder, and the destruction of anatomical outlines. Biliary calculi lying in the common duct, in the hepatic or at the mouth of the cystic duct ; stricture of the ducts from local ulceration (exceedingly rare), or occlusion of them from external causes, as cancer of the pylorus or the last portion of pancreatic duct, all require treatment for relief beyond either cholecystotomy or cholecystectomy.

I believe it is possible to freely loosen adhesions that have formed in the region of the gall-bladder, and in all cases of severe traumatisms we should not hesitate to make use of the tamponade of iodoform gauze and drainage, or employ the method so clearly recommended by one of our Fellows, Dr. Morris.

Dr. H. O. Marcy's paper on Relief for Biliary Obstruction defines the steps of the operation very closely, particularly in relation to suturing of the incision made in the gall duct.

The operative technique may be varied as follows, by—

1. Dislocation of the calculus *en masse*, either into the duodenum or into the gall-bladder.

2. Cholelithotrity, either by crushing through the walls of the duct with padded forceps or fingers, or from within the gall-bladder by means of the needle or fine probe, followed by removal by the way of the gall-bladder or intestinal canal.

3. Breaking of the calculus by the introduction of strong needles through the walls of the duct, and subsequent dislodgment of the fragments.

4. Cholecystenterostomy according to Von Winiwarter, or modifications of it as have been suggested to Gaston in his elaborate experiments upon dogs.

5. Incision of the gall duct and removal of the calculus, with subsequent suturing of the incision in the duct.

The application of these methods conflicts with some notions in surgery which to my mind are altogether fallacious. The first is, that sutures can never be applied to the gall bladder and ducts with safety. The first cholecystotomy was followed by suture and recovery.

The second notion is that the healthy biliary secretion always causes peritonitis when free in the abdominal cavity. The experience of Schuppel, Bostroun, the cases of Paraisse, Sabatier, Thiersch, etc., demonstrate the falsity of this dogma.

We must put these fallacious ideas behind us, as we have many concerning the technique and management of abdominal section. I believe in a wholesome fear of the peritoneum and of throwing around our patients every possible safeguard. I should not like to complete an operation knowing that bile might flow over the intestines, but I believe that such wounds, by proper drainage and iodoform-gauze tamponade, can be made comparatively safe for the patient, much safer than the condition for which the operation was done.

Those cases which present the least difficulties are where the calculus can be removed *en masse* by pushing either into the gall bladder after cholecystotomy (preferable) or into the duodenum. The danger of tearing the duct across its diameter, as has already occurred, must be kept in mind, and will somewhat circumscribe the operation of fracture or incision and removal from the intestine.

The second procedure, together with the third, may be employed in suitable cases, but the choice will lie between them and the fifth procedure, that is, incision and removal.

The fourth method of treatment, by establishing a new communication between the biliary ducts and the intestine, must always have a certain utility, especially where it is found that the seat of obstruction (the common duct) is bound down by adhesions and cannot be made accessible.

To one doing abdominal work I would advise a careful perusal of Dr. Gaston's paper in *The Atlanta Medical and Surgical Journal* in 1884, entitled Experimental Cholecystotomy.

In France the operation bearing the name of Von Winiwarter has recently received much attention, and flattering results are reported.

Under the fifth division—that is, incision and suture of the gall ducts—we have, I believe, in many cases a method which promises great success, where heretofore cases have been treated by establishment of biliary fistula, which only relieved for a short time. So far as

I have been able to learn, incision of the common duct has been seldom performed in this country. The technique of the operation is as follows : Usual vertical incision, from tip of cartilage of tenth rib, made of sufficient length to permit free examination. It may be necessary to complete the operation by making a transverse incision through the right rectus abdominalis. Incision over stone, in line of duct, is made. Fluid behind duct should be withdrawn by aspirator, or sponges placed to protect surrounding parts. First row of sutures continuous, introduced just within serous coat of duct, brought just within the mucous coat, but not involving it ; second series, Lembert, bringing the serous coat into accurate apposition. Surround drainage-tube on all sides with iodoform gauze tamponade, the ends being left in abdominal wound, closing latter with silkworm-gut. One or two silkworm-gut sutures may be introduced and tied in loop, so that after removal of tamponade they may be tied and abdominal wound more completely closed.

DISCUSSION OF THE TWO PRECEDING PAPERS.

Dr. R. T. Morris, of New York : There is one method of procedure, in cases of gall stones, so simple that I wonder that anybody has failed to think of it. Gall stones can be dissolved very easily by chloroform, ether, and some of the marsh-gas series. We do not need any forceps. We can remove the greatest element of danger by dissolving them right in place. The operation consists in suturing the gall bladder to the abdominal wall, then waiting for forty-eight hours until adhesion has taken place. The cases of greatest danger after operation are those followed by leakage of bile or mucus or the fermenting contents from the gall bladder into the abdominal cavity. Therefore, the ideal procedure consists in first suturing the gall bladder to the abdominal wall, waiting until adhesion takes place, opening the gall bladder, and with a syringe injecting down upon the gall stones, at any time you please, a week or a day or a month after, a chemical that will make a solution of the cholesterin quickly and safely without imposing the grave danger upon the patient of crushing, bruising, or injuring the common duct. I am making experiments in this line to find some non-irritating solution which will dissolve the gall stones easily.

Dr. L. S. Pilcher, of Brooklyn, recognized the fact, as all surgeons do, that the gall bladder has become a fit subject for surgical interference, and that, with the rapidly increasing experience which is being gained in work upon that organ, we shall soon have well-established indications for not only examining the organ, but also for the different classes of operations which we shall be called upon to do, for the relief of the conditions which we find present in it. The ideal cholecystotomy, in which the organ is exposed, is opened, is evacuated, is closed and dropped back into the abdominal cavity, suggests itself as an operation extremely desirable to be done, if the conditions are such as to make it feasible to do it. The extirpation of the gall bladder has been done, and will at times be found necessary if we are to relieve the conditions which have made operation of any kind necessary. The difficulties which attend the operation are great, and it can only be rarely that a surgeon will feel justified in undertaking its accomplishment. The opening of the common duct and the removal therefrom of a gall stone have been described to us this afternoon. The successful performance of the operation has again and again been demonstrated to us. The possibilities of relief which are open by means of that should always be present in the mind of the surgeon. He then presented and discussed some specimens of gall stones.

Dr. M. B. Ward, of Topeka, stated that he had done the operation twice on a dog, removing the gall bladder entirely. His first operation was a failure, death occurring from general peritonitis on account of some defect in the operation. The next dog got well and very fat, and was subjected to three other operations on the intestines. He inquired whether the gentlemen found it easy to bring the gall bladder up and attach it to the parietes. He found it difficult unless the gall bladder was enlarged.

Dr. Seymour, closing the discussion on his part, said, with regard to the attachment of the gall bladder to the abdominal wound, the method pursued by Mr. Tait is very satisfactory, particularly in cases of contracted gall bladder. That is, not to attempt to bring the gall bladder up to the level of the skin, but to suture the gall bladder with an interrupted buried silk suture at an intervening height in those tissues, taking a sufficient number of interrupted sutures to give strong and firm coaptation of the structures. He considered the matter of dissolving gall stones within the gall bladder as still *sub judice*. In view of that, the operation of Tait—opening the gall bladder with establishment of a fistula—is the most rational operation. He considered it very possible that there would be a recurrence of the disease with the persistence of the constitutional condition. He considered the silk suture preferable in this operation to any of the animal sutures.

Dr. Vander Veer, closing the discussion on his part, said that in reference to attaching the gall bladder to the parietes, he would only add this, that before the gall bladder is attached to the incision, and before it is opened, we should make a very careful examination of the common duct and be very certain as to the condition of the pancreas.

Dr. W. H. Wathen, of Louisville, read a paper on

### ASEPSIS IN INTRAPERITONEAL SURGERY.

If the proper precautions as regards cleanliness in every detail before and during an operation are observed, we need no antiseptic germicides in intraperitoneal surgery. If solutions of sublimate, carbolic acid, etc., are brought in contact with healthy peritoneum, their action is harmful. I will not condemn the use of chemical solutions for the purpose of sterilizing the operator, assistants, nurses, or patient, or the room, instruments, sutures, dressings, sponges, etc., if used before the operation is begun ; but the chemical germicide should be removed from everything that is brought in contact with the peritoneum. Unless everything is made practically clean independent of the germicide, that will not make it aseptic. It is too often true that operators who are loudest in advocacy of germicide solutions are the least cleanly, and I have known a few of them to forget to wash their hands before beginning an operation or before examining a woman in labor.

There are relatively few men who know how to be surgically clean in every detail connected with intraperitoneal surgery, and if the time and labor that have been devoted to teaching the medical profession how to use antiseptic germicides had been directed to teaching the value of, and means of accomplishing, surgical cleanliness, septic peritonitis following laparatomy would be comparatively infrequent. Of course, the above does not apply to all men who use chemical antiseptics, for some of them are the most cleanly men I have seen operate, but I believe they would get as good or better results if they omitted the antiseptics. The peritoneum is usually infected by contact, and the danger of atmospheric infection is practically *nil*, as has been shown by the excellent results in laparatomies done in large and crowded amphitheatres.

He explained in detail the latest and most improved methods in asepsis. He advocated drainage with a small glass tube open at both ends, with fine holes on sides extending within two inches of the mouth. He opposed the practice of introducing the wick or gauze in the tube, and preferred to remove the blood and secretions by suction with a long-nozzle syringe or a syringe with a gum tube attached.

Dr. Henry O. Marcy, of Boston, read an essay entitled

### FEMORAL AND VENTRAL HERNIA IN WOMEN.

The methods by which the author obtains a radical cure are original, and are followed by most exceptional results. He advocates the dissection of the sac to its very base, which is sutured across and removed. The internal ring is carefully closed by a line of deep, double, continuous tendon sutures. The canal is narrowed and closed in a similar manner, and the wound is sealed with iodoform-collodion, without drainage. The operation is conducted with the strictest antiseptic care; and since Dr. Marcy was the first to use and publish the advantages to be derived from buried animal sutures, and systematically to extend their applicability in the general field of surgery, we quote his emphatic directions: "There is but one rule, and it cannot be too rigidly enforced—the *aseptic* suture must be *aseptically* applied in *aseptic* structures, and the wound must be maintained *aseptic*. The failure of either of the above-mentioned factors not alone endangers the result, but may be followed by the most serious consequences. Modern surgery demands of the operator every safeguard to insure an aseptic wound, but he who uses buried animal sutures must take, if possible, even greater precautions, since infection carried into a wound thus firmly closed is, for obvious reasons, attended with much greater danger than in a wound united by interrupted sutures which, at the end of a few days, are to be removed, and where drainage is relied upon to permit the escape of infective or foreign material. It is in part on account of defective technique, the use of drainage, and the too often septic wound that failure to effect a cure after hernial operations so generally occurs.

"I began to use the buried animal suture in operating for the cure of hernia in 1871, and since that time have for the most part used it in the closure of all operative wounds; and in all my operations for the cure of femoral hernia, where the integrity of the intestine has not been involved, I have never observed a subsequent symptom indicating danger, and, so far as I have been able to learn, there has not been a single recurrence. There is little pain, and even edema of the tissues does not ensue. After a few days in bed the patient is allowed to sit up. In some instances I have permitted the use of the chair the second day, without any apparent harm. I never advise the subsequent application of a truss. . . .

"If it can be demonstrated that femoral hernia is curable, then the advisability of the operation should be taken into consideration; and if it can be proved that the cure remains permanent, it adds much to the argument in favor of operative measures; but where it is demonstrated that, under proper precautions, based upon an accurate anatomical knowledge of the structures involved, the operation is not severe, does not cause long detention from active duties, does away with the punishment inflicted by the life-long wearing of a truss, and is almost without danger, there remains no reason why all the sufferers from femoral hernia should not profit by surgical measures and demand to be restored to the ranks of active service."

Dr. Marcy makes an equally strong plea in behalf of surgical intervention for the cure of sufferers from umbilical and ventral hernia. In umbilical hernia he dissects the peritoneal sac quite within the margin of the ring, sutures it across at its base, and resects it. The subsequent steps of the operation are conducted under irrigation. There are conditions when it is wise to resect the ring and close as in an ordinary laparatomy; but the method which Dr. Marcy more generally recommends, is one quite peculiar to himself. The structures composing the ring are divided laterally upon the plane of the abdominal wall, about one-half of an inch in all directions. This admits the coaptation of the sundered parts, and, by lines of strong, continuous tendon sutures, the separated edges are coaptated in a way greatly to broaden the united parts. This widens the line of union to an inch or more, instead of bringing together the narrow edges of the tendinous ring; and, besides affording this great depth to the united portions, it brings together refreshened surfaces in a high state of vitalization, likely to be followed by firm union. It also admits the joining of the tissues in three distinct layers of strong sutures. As in the other forms of hernia, the skin itself is closed by a line of running or lacing sutures, taken from side to side through the deeper portions of the skin only, which admits of its coaptation by sutures entirely hidden from view. Such a wound requires no drainage, and it is permanently sealed with collodion. The weight of years of experience in active surgical practice enforces the value of Dr. Marcy's contributions upon a subject of the highest surgical importance, since the radical cure of hernia is considered by many as yet *sub judice.*

### DISCUSSION.

Dr. J. H. Carstens, of Detroit, said everything stated in the paper gave him pleasure except the employment of ether. He hoped some time the hub would learn from the tire out West that chloroform was the best anesthetic. In operating he made the usual incision, and returned the gut if all was right. He then took silk, with the needle which he used for operating on the lacerated cervix, and encircled the hernial opening completely. He tied that so as to close it up perfectly. He did not like to trust the buried animal suture. He dissected out the sac in the usual manner and cut it off short, then took catgut and with that sewed the wound carefully in the usual manner until the wound was entirely closed except the skin, which he closed with two or three silkworm-gut sutures. He used no drainage-tube. He wished to strongly emphasize what Dr. Marcy had insisted upon, viz., that the time for the drainage-tube in the operation for hernia ought to cease.

Dr. H. O. Marcy then exhibited to the Association some specimens of kangaroo tendon, and explained the method of preparing the various animal sutures.

Dr. W. J. Asdale, of Pittsburgh, said, with reference to the drainage-tube, that he used it when in doubt what to do. He regarded the tube with many small perforations as a dangerous one. He followed the practice, where he used the drainage-tube, of turning it at short intervals, every few hours—at any rate, at every visit.

Dr. R. B. Hall, of Cincinnati, said that he had practised drainage in every case of abdominal section since October, 1887. He had never had occasion to regret placing the drainage-tube. Some cases in which he placed the tube, feeling at the time that it was not necessary, would, undoubtedly, have died had the tube not been placed.

Dr. E. E. Montgomery, of Philadelphia, had for a long time been averse to introducing silk either into the wound or covering it up in the abdominal cavity. In re-operation upon a case a year or more after the first operation, he had found the silk still remained, and in some cases it was a source of irritation. He was strongly inclined to the use of the animal ligature. He had not had the opportunity to use kangaroo tendon, but had resorted to carefully prepared catgut. He considered the care of the instruments as a very important part of the technique of abdominal surgery, particularly the needles. He advocated cleansing the needle with a soapy towel, then cleaning it with benzine, and finally placing it in absolute alcohol. When the catgut and needles were ready for use, they were placed in a tray and covered with alcohol. If the catgut is placed in water, it softens and swells, and is somewhat difficult to tie ; but from the alcohol it is hard, and when it is introduced, it swells and fills up the track made by the needle. Dr. Wathen made a good point in regard to drainage. It is usually considered that drainage should be from the lowest portion of the abdomen ; but if we look at the abdomen when it has been cleansed and the intestines have settled down, we will find that the liquid gravitates to the upper surface. He had no doubt but that many cases of intestinal adhesion were due to the fact that there was present a fluid which, to a slight degree, became infected sufficient to give rise to a slight superficial peritonitis and gluing together of the surface. In these cases drainage by gauze or by wick is oftentimes very efficient.

[*Concluded next month.*]

